# Clinical Decision Support Systems for Comorbidity: Architecture, Algorithms, and Applications

**DOI:** 10.1155/2017/1562919

**Published:** 2017-03-08

**Authors:** Aihua Fan, Di Lin, Yu Tang

**Affiliations:** ^1^Xi'an Polytechnic University, Shaanxi, China; ^2^University of Electronic Science and Technology of China, Sichuan, China

## Abstract

In this paper, we present the design of a clinical decision support system (CDSS) for monitoring comorbid conditions. Specifically, we address the architecture of a CDSS by characterizing it from three layers and discuss the algorithms in each layer. Also we address the applications of CDSSs in a few real scenarios and analyze the accuracy of a CDSS in consideration of the potential conflicts when using multiple clinical practice guidelines concurrently. Finally, we compare the system performance in our design with that in the other design schemes. Our study shows that our proposed design can achieve a clinical decision in a shorter time than the other designs, while ensuring a high level of system accuracy.

## 1. Introduction

A clinical decision support system (CDSS) is designed for ubiquitous health monitoring, which provides mobile health service in order to help physicians and doctors monitor patients' conditions anytime and anywhere with the aid of wireless networks [1]. In such an ubiquitous health monitoring system, building on the biosignal data of patients, a CDSS can assess their conditions with a certain algorithm and inform physicians and doctors immediately when an emergency case occurs, being able to reduce the delay for medical treatment of a critical patient [2].

Most work in the design of CDSS has focused on making clinical decisions on one type of disease, either following the clinical guidelines of this specific disease or employing various data mining schemes to extract clinical rules from numerous biosignal data of patients [3–10]. The primary algorithms include *k*-nearest neighbors based algorithms (*k*-NN), artificial neural networks based algorithms (ANN), decision trees based algorithms (DTs), fuzzy logic based algorithms (FL), and the extension of these algorithms by merging them.

*(a) K-Nearest Neighbors Based Algorithms*. A *k*-nearest neighbors based algorithm is a method for classifying cases by referring to the closest training cases in the feature space. A typical *k*-NN algorithm runs as follows [10]: cases in question are firstly mixed with the other known cases to constitute a case set. Next, all these cases in this set are mapped into a finite-dimension feature space. A dimension in this space characterizes a feature of cases, so a point in this space uniquely represents a case. Finally, all the points in this space are classified into *k* disjoint subsets, and each subset represents one type of cases. The type of cases in question is assigned by the type of known cases which are within the same subset.

*(b) Artificial Neural Network Based Algorithms*. Quite similar to the structure of a human brain, an ANN based algorithm is composed of a few interconnected layers of neurons and extracts the exact way of connecting layers by training the historical data of patients. Based on the different training schemes, numerous ANN methods are presented in the literature. In the context of wireless health, two main ANN based algorithms are widely employed in a CDSS [9], including clustering, self-organizing map: clustering based training scheme is implemented by extracting a few critical features of cases and then mapping these features into the structure of an ANN [8]; self-organizing map alters the structure of an ANN by adding necessary neurons and deleting unnecessary neurons [7].

*(c) Decision Tree Based Algorithms*. Generally, a decision tree based algorithm makes a decision through a series of question and answer sessions. Firstly, the tree based algorithm starts with a root node, which represents the most significant feature in determining the condition of a patient. Then, cases with different states of this feature are split into two or more branches. If all the cases fall into one branch, the branch can be denoted as a leaf. The process of splitting is repeated until all the branches are denoted as leaves [5].

*(d) Fuzzy Logic Based Systems*. The application of fuzzy models into decision support systems is motivated by the uncertainty of thresholds to make a decision on the patient condition [4]. For example, if the normal range of heart rate is 80–90 times/min, a heart rate of 79 or 91 times/min should not be certainly determined as abnormal [3]. Instead, the result should be determined as normal with a certain probability. In general, the fuzzy process is composed of three procedures: (1) Fuzzification, which is characterized as mapping crisp inputs into fuzzy variables by acquiring the membership values of these crisp inputs, the membership functions can take different shapes, and the simplest but quite widely employed is trapezoidal function; (2) inference based on some rules; (3) defuzzification, which is characterized as converting fuzzy variables into crisp outputs with various mapping methods, and one typical defuzzification method is the centroid of area (COA) method.

Also a few CDSSs are designed to implement in the scenario of making clinical decisions on a comorbid patient (e.g., a patient with Atrial Fibrillation in the setting of Wolff Parkinson's White Syndrome), and in such scenarios the decisions on the treatment of multiple diseases might be in conflict with each other. Such a need is especially clear for elderly patients: several studies have shown that about half of people above 65 years old have a comorbid condition [11]. Boyd et al. in [12] discuss the employment of multiple clinical practice guidelines (CPG) to the care of patients in a comorbid condition. By analyzing various hypothetical clinical scenarios of typical clinical diseases and their associated CPGs, the authors conclude that a blind combination of individual CPGs in the presence of comorbid condition may negatively impact a clinical decision due to the potential mutual conflicts between guidelines. They explain this situation by the fact that a CPG is developed by a specialty-dominated committee and thus is not designed to provide help with regard to evaluating and managing comorbid patients. The conclusion in [12] motivates the research on how to develop a computing algorithm in the design of CDSS for making clinical decisions on the patients in a comorbid condition. Research in the stream of computing algorithms in the design of CDSS for patients in a comorbid condition, despite its importance, seems to be in its infancy. Riaño et al. in [13] advocate using an ontological model for guideline personalization. Unfortunately, the development of this model needs to be conducted manually by physicians using a specialized clinical guideline editor instead of automatically by a computer. In [14], the authors propose a methodology as well as its specific implementation for automatically combining multiple clinical guidelines; however, the data of patients need to be manually entered by physicians.

### 1.1. Motivation

All these above-mentioned papers do not address how to establish a system which can automatically extract the patient data and offer a decision for clinical practice. Also all the aforementioned research focuses on the level of algorithms and do not mention the design of a CDSS at the system level. The importance of designing a CDSS for comorbidity monitoring as well as the lack of efficient design in the system level motivates us to investigate how to design a CDSS for monitoring comorbid patients by merging the clinical guidelines as well as how to assess the system performance.

### 1.2. Main Contributions

In this paper, we present the design of a CDSS for monitoring comorbid conditions, specifically from the perspectives of the architecture, the algorithms, and the applications of a CDSS. Also we address how to analyze the accuracy of a CDSS in consideration of the potential conflicts when using multiple clinical practice guidelines concurrently.

The primary contributions of this paper rest at the following issues:

- address the architecture, the algorithms in the design of a CDSS for comorbidity;
- present the implementation process of a CDSS for comorbidity;
- assess the system accuracy of a CDSS for comorbidity.

## 2. Architecture of CDSS for Comorbidity Monitoring

**Figure 1:**
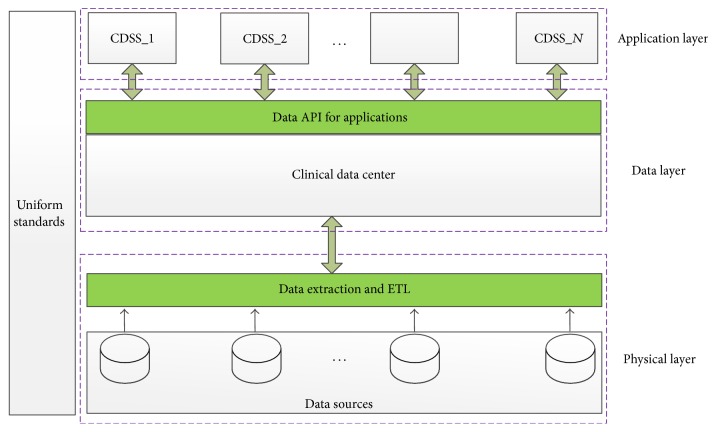
Architecture of a CDSS for comorbidity monitoring.

In this section, we address the architecture of a CDSS in our design for comorbidity monitoring, and it is shown in Figure 1. As shown in Figure 1, the architecture of a CDSS is composed of three layers: physical layer, data layer, and application layer.

**Figure 2:**
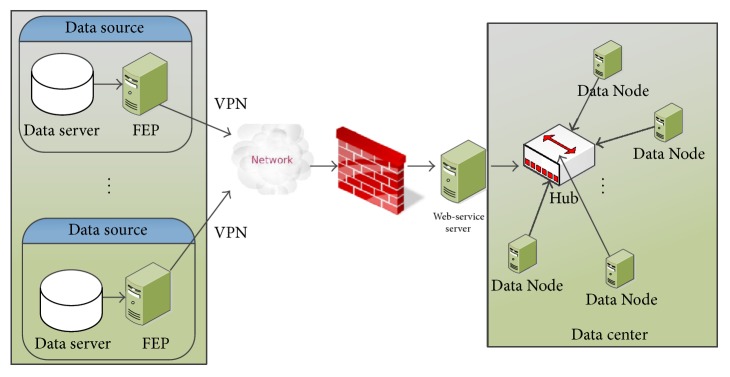
Physical layer of a CDSS.

In the physical layer, the system needs to extract data from various data sources. The primary data source in clinical scenarios is hospital information systems (HIS), which are located in hospitals or medical centers. Also the medical data may be from a few medical sensors, including the medical sensors attached to or even embedded into the body of a patient, for example, Holter and blood pressure sensors. As shown in Figure 2, data from different sources might be in different formats, and we employ a front end processor (FEP) to do the data extraction, transformation, and loading (ETL) process and also convert data into XML or JSON. In consideration of the security issues, we add a virtual private network (VPN) connection into the system, and also we use a firewall and web service server at the end of data center, to ensure that the data center is not directly connected to public networks for security issues.

**Figure 3:**
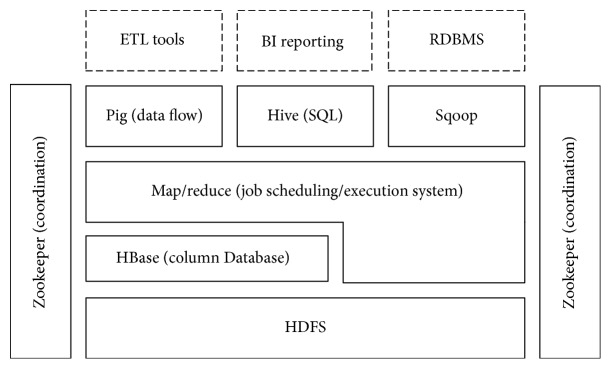
Hadoop ecosystem.

In the data layer, with the dramatic increase of clinical data, the traditional data processing applications are inadequate to process such a large set of data, so we employ a Hadoop/Hbase based server cluster to store and process data. The data stored in the data center are prepared to offer data APIs to the application layer, and we use a few off-the-shelf tools of Hadoop to achieve the data APIs [15]. The detailed Hadoop ecosystem is shown in Figure 3. Specifically, we employ Pig and Hive to support the application layer by combining them with Hbase [16] and also Sqoop to connect with the traditional relational databases.

In the application layer, the system can support the CDSS for specific clinical diagnosis, and we focus on the diagnosis on the comorbid patients. The CDSS for comorbidity diagnosis needs to merge clinical practice guidelines (CPG) and detect and mitigate the potential conflicts between CPGs. After mitigating all the conflicts, the system can achieve a final decision on the clinical practice. In the design of an application layer, we use a constraint logic programming model to represent all the CPGs and detect the potential conflicts. The detailed algorithms are illustrated in Sections 3 and 4.

Additionally, the issue of security is critical in the application of healthcare, and generally the security issues in Hadoop involve three aspects: authentication, authorization, and network encryption. We can employ a few off-the-shelf security solutions in the architecture of Hadoop. A summary of security solutions in the Hadoop for security is presented as follows.

*(1) Authentication*. Kerberos is a secret-key based authentication system, which can provide authentication and Single-Sign-On (SSO) of users and servers in a network. Hadoop uses the Kerberos protocol to authenticate users to Hadoop and to authenticate Hadoop services to each other.

*(2) Authorization*. Hadoop provides authorization controls to authenticated users with the aid of HDFS file permissions and service-level authorization. Each of the files is associated with a group and has associated permissions of read and write. Based on such file permissions, HDFS controls access to reads and writes to the distributed file system. Service-level authorization is used by a cluster to control the permission of users to submit jobs.

*(3) Network Encryption*. If a few sensitive data are exchanged in a network, then we need to protect the cluster from eavesdroppers and network sniffers by encrypting these data.

## 3. Algorithms of CDSS for Comorbidity Monitoring

As shown in Figure 1, the architecture of a CDSS is composed of three layers: physical layer, data layer, and application layer. In this section, we present the specific algorithms in each of the layers.

### 3.1. Physical Layer

**Figure 4:**
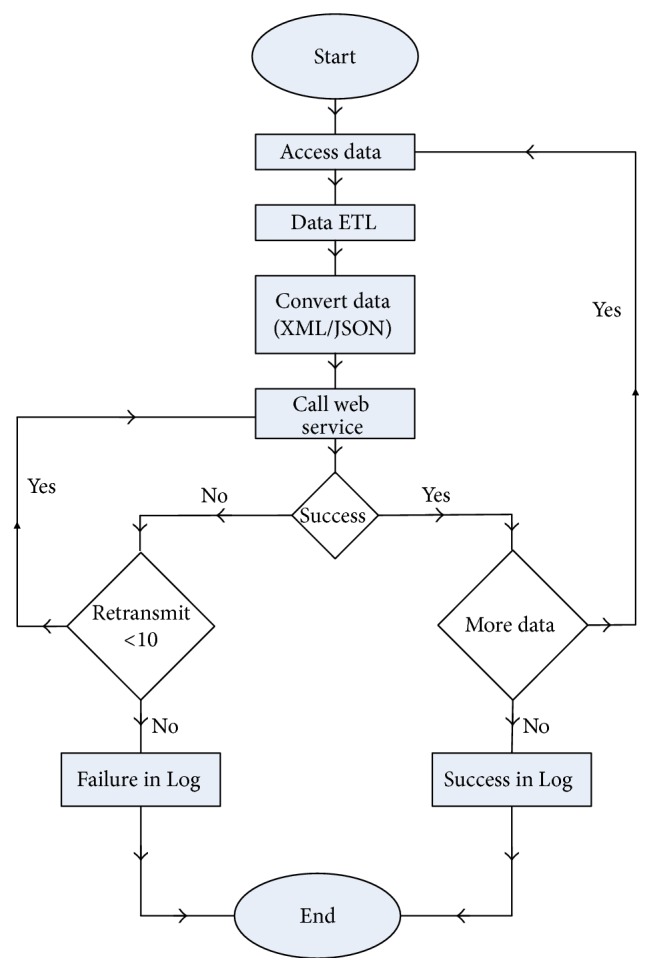
Algorithms in physical layer.

In the physical layer, the CDSS extracts data from various data sources and runs the ETL on raw data in order to load the processed data (clean data) into the data center. The detailed algorithm in the physical layer is in Figure 4. The CDSS firstly accesses the data from data sources and extracts data, does the data cleaning, and standardizes the data into the XML or JSON format. Data cleaning includes data transformation, duplication elimination, replacing missing data, and removing outliers. Of course, the process of data cleaning refers to a standard, for example, HL7, and thus the system supports HL7 to transform the data from different sources (in different formats) into the data in a uniform format. Then, the CDSS calls a web service, and once the connection is successfully built up, the formatted clean data would be transmitted to the data center. Usually, the data are transmitted in bulk, so the data transmission would finish until all of the bulks of data are transmitted. When calling the web service, if the connection is failed, the CDSS would recall the web service 10 times and write the results into the Log if none of 10 tries succeeds. Otherwise, when the connection succeeds, the CDSS would extract more data and repeat the above-mentioned procedures.

### 3.2. Data Layer

In the data layer, the clinical data are stored in Hbase tables, the design of which is shown in Table 1. In the CDSS applications, we consider the big data computation architecture and thus employ Hadoop architecture. Hbase is the native database of Hadoop and is well compatible with the tools of Hadoop ecosystem. In comparison with the relational database, Hbase can improve the computation performance, that is, running efficiency. As shown in Table 1, Hbase tables are composed of row keys, time stamps, and column families. A row key is the unique key in a Hbase table and it can be used for indexing in the Hbase table. In the scenario of clinical data, a row key is represented by the patient ID. Time stamps are automatically generated by Hbase, and they denote the time of data entry. Column families are usually a set of columns, and one column represents a property of patients. For example, in the first row, the row key “11112222” represents the identification (ID) of such a row. The time stamps “T7,” “T8,” and “T9” represent the time of data entry. Specifically, the entry of “Name = Peter” in the column “PersonBasicInfo” and the entry of “National = Canada” in the column “PersonExternalInfo” occur at the time stamp of “T9”; the entry of “IDNumber = 11112222” in the column “PersonBasicInfo” and the entry of “FamilyDisease = None” in the column “PersonExternalInfo” occur at the time stamp of “T8”; the entry of “Address = Ottawa” in the column “PersonBasicInfo” and the entry of “Allergen = None” in the column “PersonExternalInfo” occur at the time stamp of “T7.”

### 3.3. Application Layer

In the application layer, the algorithms of our CDSS are developed with CLP-CPG models. A CLP-CPG model refers to the development of a combined clinical practice guideline for a specific patient with the aid of constraint logic programming (CLP) [14]. The CLP-CPG model is motivated by the fact that the diagnosis and treatment plan for a specific patient may not be made in consideration of only one CPG; instead, the consideration of combining multiple CPGs is necessary to develop a treatment for patients, especially for those with comorbidity [17].

**Figure 5:**
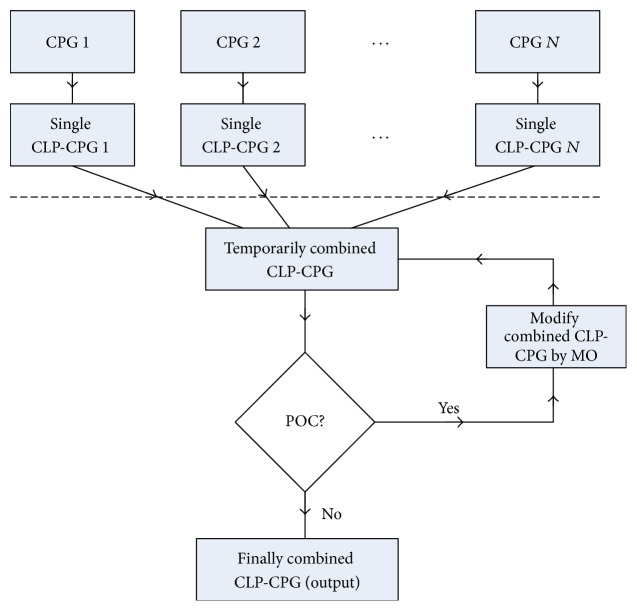
Flow chart of a CPG-CLP model given *N* CPGs to combine (CLP = constant logic programming, CPG = clinical practice guideline, POC = point of contention, and MO = mitigation operator).

However, the challenge of combining multiple CPGs is the possible conflicts among multiple CPGs, these conflicts constituting a point of contention (POC) [18]. The CLP-CPG model attempts to mitigate all the POCs by modifying the details of a CPG with the approval of physicians, and the modification of CPGs leads to a modified CLP-CPG model. The modified CLP-CPG model can provide a complete diagnosis and treatment plan to physicians if all the POCs are mitigated. Otherwise, physicians can achieve the conflicts between the CPGs, and they will manually make a complete plan based on the information offered by the model. The work flow of a CLP-CPG model can be shown in Figure 5, given that *N* CPGs are considered.

In the following, we show the detailed process of establishing a CDSS for comorbidity monitoring by providing the example of CDSS for both hypertension (HTN) and deep vein thrombosis (DVT), but our design of CDSS for comorbidity is applicable to the monitoring of comorbid patients with other diseases. The work flow is composed of 3 steps (see details in Section 4): (1) For each of CPGs (in the format of text or graph), the system can establish a corresponding CPG-CLP model to represent the specific CPG. (2) Usually, the CDSS for comorbidity requires multiple CPGs, which might be in conflict with each other. Thus, the second step is to combine each of the CPG-CLP models which are relevant to the comorbid patient and investigate the potential conflicts. (3) When any conflict exists, the CDSS needs to find a solution which can mitigate the conflict. If no conflict exists, the CDSS can output the final plan of making diagnosis decisions on the patient; if all the possible solutions are tried, but cannot mitigate all the conflicts, the CDSS needs to report the conflicts to the physicians. Then, the physicians need to manually make a plan, and the CDSS memorizes the solution by the physicians for future decision making.

## 4. Applications of CDSS for Comorbidity Monitoring

In this section we present how to use a CLP-CPG model in a clinical scenario for comorbidity monitoring. Specifically, we take the scenario of a patient suffering from both hypertension (HTN) and deep vein thrombosis (DVT) as an example. In such example, we use anonymized patient data transcribed from a public data source [19]. Please note that the design of a CDSS for HTN/DVT monitoring can also be applied to the monitoring of the other diseases.

### 4.1. Clinical Guideline

**Figure 6:**
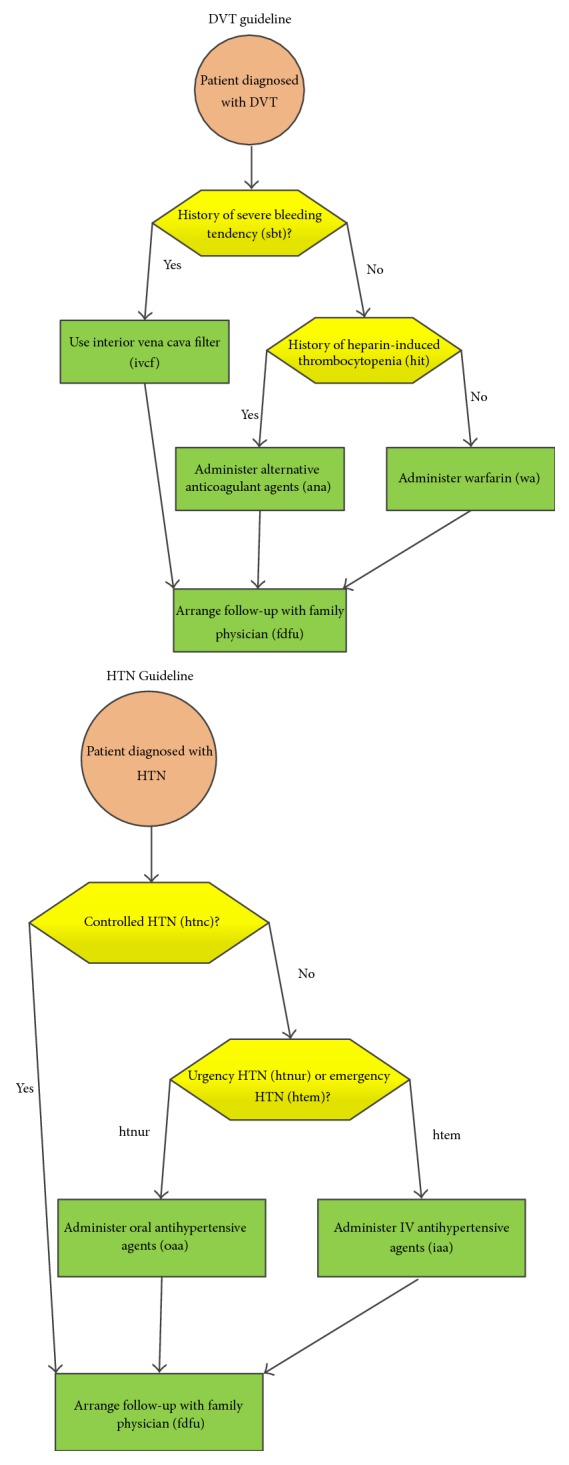
Clinical guidelines for HTN and DVT [17].

The clinical guidelines for a patient who has both HTN and DVT are illustrated in Figure 6. Based on the clinical guideline of DVT, the patient would be inquired whether he or she has a history of severe bleeding tendency (sbt). If so, a physician would recommend this patient to use an inferior vena cava filter (ivcf). Otherwise, this patient would be inquired whether he or she has a history of heparin-induced thrombocytopenia (hit). If so, this patient would be required to use anticoagulant agents (ana), and otherwise this patient would be recommended to take warfarin (wa). Finally, after this patient uses ivcf or ana or wa, the physician would transfer this patient to his or her family doctor to follow up (fdfu).

Based on the clinical guideline of HTN, a patient would be checked to confirm that the hypertension he or she suffers is controlled (htnc) or uncontrolled (htnuc). If the type of hypertension is in the type of controlled, the patient would be directly transferred to his or her family doctor to follow up. If the type of hypertension is uncontrolled, the patient would be further checked to confirm whether this patient's hypertension is in urgency (htnur) or emergency (htnem). If the patient is in the status of htnur, the patient would be required to take oral antihypertensive agents (oaa) and otherwise to take the IV antihypertensive agents (iaa). Finally, the patient would be transferred to his or her family doctor to follow up. For a patient with both HTN and DVT, he/she must follow the guidelines of both. An adverse interaction between guidelines for HTN and DVT is that a patient who suffers urgency hypertension (htnur) cannot take warfarin (wa) or anticoagulant agents (ana), since wa and ana have negative impact on the treatment of hypertension. In such a case, physicians must replace the wa or ana with the other types of treatment, that is, intra-vena cava filter.

### 4.2. Work Flow of a CDSS for Comorbidity Monitoring

In this section, we would like to address the detailed design of a CDSS, and usually a CDSS is embedded in a patient device. As the primary component of a patient device, a CDSS is designed by referring to clinical guidelines and can output the diagnosis on patient conditions. The first procedure of designing a CDSS is to combine multiple clinical guidelines by translating each of the clinical guidelines into a logic expression. A clinical guideline is composed of a few clinical rules, and each of the rules can be represented by a logic programming expression. Thus, we can use the constraint logic programming to combine clinical practice guidelines by following the 3 steps: (1) Translating the rules of each clinical guideline into a few logic expressions; (2) combing the logic expressions of multiple guidelines together; (3) transforming the constraints between all of the guidelines into logic expressions.

*(1) Transform Rules in the Clinical Guidelines*. A clinical guideline consists of multiple clinical rules. For each rule in a clinical guideline, we can denote it in the form of a logic expression. For example, we can transform the rule of sbt → ivcf → fdfu into the logic expression of sbt∧ivcf∧fdfu; we can transform the rule of htnc → fdfu into the logic expression of htnc∧fdfu. Similarly, we can transform all of the rules in clinical guidelines into logic expressions.

*(2) Transform Constraints between Guidelines*. Taking warfarin (wa) or anticoagulant agents (ana) could result in negative impacts on an urgency hypertension patient. Thus, we need to set a constraint of htnur for the combination of guidelines of HTN and DVT. In this paper, we assume that the constraints are manually analyzed and set by a few physicians. In the future, when all these constraints, that is, the conflicts between clinical guidelines, can be analyzed and stored in a repository, the CDSS can automatically detect the potential conflicts by searching in the repository. This constraint is not presented in any individual clinical guideline but appears when we need to combine multiple guidelines together for the diagnosis on a comorbid patient. In the form of logic expressions, we can denote this constraint as not  (htnur∧wa) and not  (htnur∧ana). When this constraint is violated, that is, in the setting of htnur∧wa, the clinicians must adjust the regular treatment plan for this patient.

### 4.3. Establish a Constraint Logic Programming Model

Based on Sections 4.1 and 4.2, we can establish a constraint logic programming (CLP) model by transforming rules as well as the constraints between multiple clinical guidelines. Consequently, the problem of designing a CDSS can be characterized as a CLP model, and the detailed model is shown in Table 2. In the CLP model, we can capture the clinical decision by ensuring that at least one of the logic expressions which represent the rules of HTN and DVT is “true.”

In the stage of implementation, we employ an ECLiPSe platform [20] to represent the above-mentioned CLP model which is established by referring to Table 2. ECLiPSe is a platform designed to support a CLP modelling language—Zinc, which is high-level enough to well characterize most CLP problems and also low-level enough to be easily compatible with many CLP solvers. Also the ECLiPSe offers a special library (called repair) to capture any violations of constraints between CLP expressions. When a constraint in the CLP expressions leads to a conflict, it can be captured by the repair library.

In the following, we present a few sample ECLiPSe codes for capturing the violations of constraints between clinical guidelines:

  Constraint1 (htnur, wa) r_conflict cs1,
  Constraint2 (htnur, ana) r_conflict cs2,
  conflict_constraints (cs1, Conflicts),
  conflict_constraints (cs2, Conflicts),
  Constraint1 (htnur, wa): (htnur and wa) $ = 0,
  Constraint2 (htnur, ana): (htnur and ana) $ = 0.

To illustrate the results of the above-mentioned ECLiPSe sample codes running on the platform, we consider the following clinical scenario: a patient takes both htnur and wa; then ECLiPSe will return the results

  Conflicts = [Constraint1 (1,1)].

As expected, ECLiPSe captures the violation of constraint Constraint1  (htnur, wa) between clinical guidelines. Then, the CDSS would find a few solutions in order to mitigate the violation of constraints, for example, replacing a few types of medicine by the others. If a CDSS cannot find any solutions, the results would be reported to the physicians who will manually handle the problem.

## 5. Analysis on the System Accuracy of a CDSS

The nodes in a CLP-CPG model of a CDSS are composed of decision nodes (i.e., the yellow hexagon box in Figure 6) and process nodes (i.e., the green rectangle box in Figure 6). The errors of a CDSS can only occur at the decision nodes when the physicians make a mistake in diagnosis or recording the testing results. In such a case, the final decision made by a CDSS might also be incorrect. In this section we analyze the system accuracy of a CDSS to investigate the performance of our design of system architecture.

The system accuracy (ACC) refers to the probability of correct decision results of both HTN and DVT, and its value equals the multiplication of *P*_HTN_ and *P*_DVT_:

(1) $$\begin{array}{c} ACC=P_{HTN}\times P_{DVT}, \end{array}$$

where *P*_HTN_ represents the probability that the decision for HTN is correct, while *P*_DVT_ represents the probability that the decision for DVT is correct. Given that

(2) PDVT=Psbt=1,sbt^=1+Psbt=0,sbt^=0Phit=1,hit^=1+Psbt=0,sbt^=0Phit=0,hit^=0,

where sbt = 1 and sbt = 0 denote that the patient has a history of severe bleeding tendency or not (refer to Figure 6), respectively, while $\hat{sbt}=1$ and $\hat{sbt}=0$ denote that the physicians record that the patient has a history of severe bleeding tendency or not, respectively. Similarly, hit = 1 and hit = 0 denote that the patient has a history of heparin-induced thrombocytopenia or not (refer to Figure 6), respectively, while $\hat{hit}=1$ and $\hat{hit}=0$ denote that the physicians record that the patient has a history of heparin-induced thrombocytopenia or not, respectively.

(3) PHTNPhtnc=1,htnc^=1+Phtnuc=1,htnuc^=1·Phtnur=1,htnur^=1+Phtnuc=1,htnuc^=1·Phtnem=1,htnem^=1,

where htnc = 1 denotes that the patient actually suffers the controlled hypertension, and $\hat{htnc}=1$ denotes that the patient is diagnosed to suffer the controlled hypertension (refer to Figure 6). Similarly, htnuc = 1 denotes that the patient actually suffers the urgency hypertension, and $\hat{htnuc}=1$ denotes that the patient is diagnosed to suffer the urgency hypertension (refer to Figure 6), respectively. htnem = 1 denotes that the patient actually suffers the emergency hypertension, and $\hat{htnem}=1$ denotes that the patient is diagnosed to suffer the emergency hypertension, respectively.

The rationale for (2) and (3) is calculating the probability of correct decisions on each rule of Table 2.

## 6. Simulation and Results

In consideration of the errors in the clinical diagnosis, we investigate the system accuracy of a CDSS. Also we compare the average running time of achieving a clinical decision by the CDSS in our design with that in the other designs. In the comparison, we employ anonymized patient data transcribed from a public data source [19]. MIT-BIH database is a well-known standard ECG database, which is used universally for ECG analysis purpose. ECG signals in the database have normal sinus rhythm with a sample rate of 128 Hz and have uniform intervals of 7.8125 ms. We employ all the samples in the above-mentioned database as the dataset to analyze the performance of various systems.

### 6.1. Accuracy of a CDSS

Based on (1), the accuracy has been assessed both analytically and through Monte-Carlo simulations. In the Monte-Carlo simulations, both the actual patient status and the diagnosed patient status are generated. If the clinical decision results with both the actual status and the diagnosed status match well, then, we increase the number of correct clinical decisions by 1; otherwise, we increase the number of incorrect clinical decisions by 1. Such a process is repeated 10^5^ times. Finally, the numbers of both correct and incorrect clinical decisions are recorded. Also we can compute the accuracy of a CDSS by dividing the number of correct clinical decisions by that of all clinical decisions (both correct and incorrect decisions). For both analytical and simulation results, we define $SNR=P(x=\hat{x})/P(x\neq \hat{x})$ in the following simulation, where *x* = sbt, hit, htnc, htnuc, htnur. A large value of SNR represents a small number of errors in the clinical diagnosis.

**Figure 7:**
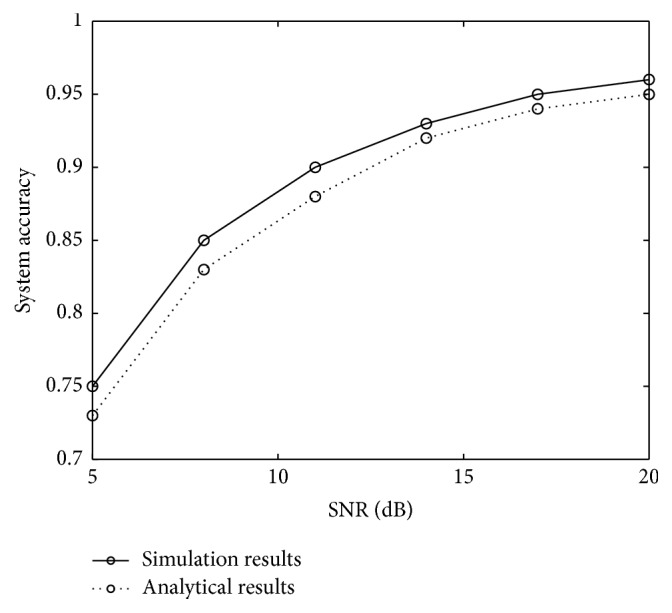
Average system accuracy versus SNR.

Figure 7 illustrates that both analytical results and simulation results of the average accuracy of a CDSS match well, verifying the accuracy of our analytical results. Also Figure 7 illustrates that our designed system can achieve a 95% of accuracy when the level of SNR reaches 20 dB or above (i.e., the average probability of diagnosis errors is around 1% or below).

As expected, we can find that the system accuracy increases with the values of SNRs. However, when the SNR is increasing, the cost of system may also increase, because it takes cost (both in time and in money) to help physicians or clinical staff reduce their errors in the diagnosis or recording the testing results. Given an acceptable level of system performance, we should search for a necessary SNR to guarantee the performance of a CDSS. For instance, we need to design a CDSS with an acceptable level of system accuracy as 95%; we must guarantee the SNR higher than 20 dB (SNR depends on the communication channel conditions between the transmitter and the receiver. In the simulation, we analyze the accuracy with the change of SNR in order to investigate the accuracy of a system under various channel conditions. However, the level of SNR cannot be manually adjusted.). Such a rule of system design can partially determine whether clinical staff or physicians need to be trained. Thus, the analysis on system accuracy can not only help healthcare staff estimate the accuracy of a system, but enable the administrator of a hospital or medical center to make the plan of training their staff.

### 6.2. Running Time of a CDSS

In this section, we compare the running time of a CDSS in our design with that in a few current designs. The running time is primarily determined by the design in the physical layer and data layer. More specifically, we investigate the impact of FEP (FEP versus no FEP) in the physical layer as well as Hbase (Hbase versus MySQL) in the data layer on the running time of a CDSS.

**Figure 8:**
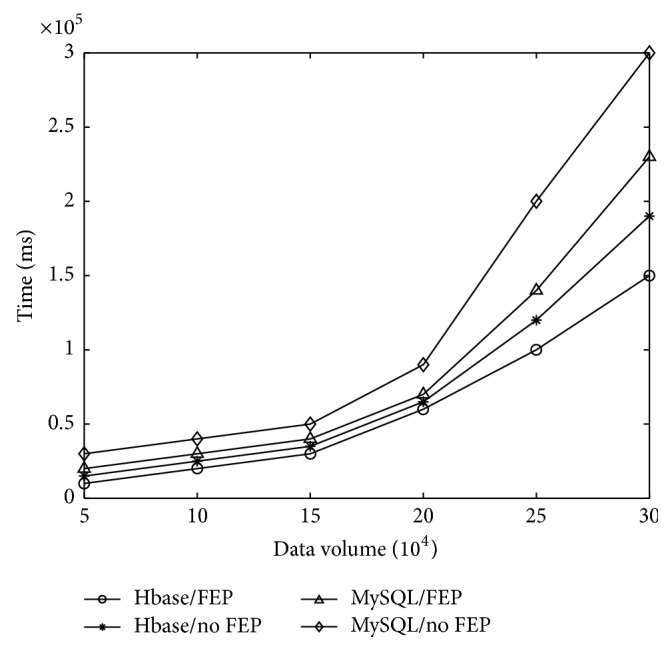
Running time of CDSS in various designs.

Figure 8 shows that the running time of a CDSS with Hbase and FEP can dramatically decrease in comparison with that without Hbase and FEP. When the data volume reaches 300000 entries (data volume refers to the number of entries in the data table. The number of entries does not mean the number of users, since a user usually has multiple entries; e.g., a patient may visit a clinic multiple times and therefore have multiple entries of clinical records. Thus, 300000 entries are possible in the real world), the running time of a CDSS with Hbase and FEP is just around a half of that of a CDSS without Hbase and FEP. Also we investigate the proportion of contributions made by the use of Hbase and FEP on the decrease of running time. Figure 8 shows that the design of Hbase in the data layer is the primary contributor, while the design of FEP makes less contributions on reducing running time than the design of Hbase. When the data volume reaches 300000 entries, the use of Hbase can achieve an 8 × 10^4^ decrease of running time, while the use of FEP can only attain a 4 × 10^4^ decrease of running time.

## 7. Discussion and Conclusion

In this paper, we present the detailed design of a CDSS for monitoring comorbid conditions from the perspectives of architecture, algorithms, and applications. Specifically, we establish the architecture of a CDSS with three layers, that is, physical layer, data layer, and application layer, and discuss the detailed algorithms in each of the layers. Also we address the application of our CDSS in a real scenario of monitoring comorbid patients with both DVT and HTN and analyze the system accuracy of a CDSS. Finally, we compare the running time of making a clinical decision in our design with that in the other designs. The results are that our proposed design of CDSS can achieve a clinical decision faster than the other designs, while ensuring a 90%–95% of the system accuracy.

Using typical clinical scenarios, we have shown how our scheme can process two clinical guidelines by developing a computable model to identify the adverse interactions between clinical guidelines. Such a scheme can support the concurrent application of clinical guidelines, and it is an important step in the process of building up an automated CDSS for the management of comorbid patients.

In the following, we present a few open issues we will work on: (1) Expanding our proposed scheme to process more than two CPGs at a time: while the proposed scheme is general enough, the increased complexity in the operations on multiple guidelines requires substantial modifications to the proposed scheme; (2) allowing for dosages of medications for a detailed description of adverse interactions: this requires revisiting definitions of logical models in our proposed scheme; (3) allowing for the automatic extraction of clinical knowledge from diversified sources to update the scheme of processing multiple clinical guidelines.

## Figures and Tables

**Table 1:** Design of table in Hbase.

Row key	Time stamp	ColumnFamily	
		PersonBasicInfo	PersonExternalInfo
^*∗*^_11112222_^*∗*^	T9	Name = “Peter”	National = “Canada”
	T8	IDNumber = “11112222”	FamilyDisease = “None”
	T7	Address = “Ottawa”	Allergen = “None”

^*∗*^_22223333_^*∗*^	T6	Name = “Steven”	National = “USA”
	T5	IDNumber = “22223333”	FamilyDisease = “Diabetes”
	T4	Sex = “Male”	Allergen = “Sea food”

^*∗*^_33334444_^*∗*^	T3	Name = “Lee”	National = “China”
	T2	IDNumber = “33334444”	FamilyDisease = “None”
	T1	Sex = “Male”	Allergen = “Peanut”

⋮	⋮	⋮	⋮

**Table 2:** CLP model for combining clinical guidelines.

Transform	CLP expression
Paths (DVT)	sbt∧ivcf∧fdfu,
	not (sbt)∧hit∧ana∧fdfu,
	not (sbt)∧not (hit)∧wa∧fdfu.

Paths (HTN)	htnc∧fdfu,
	htnuc∧htnur∧oaa∧fdfu,
	htnuc∧htnem∧iaa∧fdfu.

Constraints	not (htnur∧wa)
	not (htnur∧ana).
